# Choosing and Using Introns in Molecular Phylogenetics

**Published:** 2007-06-14

**Authors:** Simon Creer

**Affiliations:** School of Biological Sciences, University of Wales, Bangor, Gwynedd, LL57 2UW, United Kingdom

**Keywords:** introns, EPIC primers, recombination, length variant heterozygote (LVH), indel, alignment

## Abstract

Introns are now commonly used in molecular phylogenetics in an attempt to recover gene trees that are concordant with species trees, but there are a range of genomic, logistical and analytical considerations that are infrequently discussed in empirical studies that utilize intron data. This review outlines expedient approaches for locus selection, overcoming paralogy problems, recombination detection methods and the identification and incorporation of LVHs in molecular systematics. A range of parsimony and Bayesian analytical approaches are also described in order to highlight the methods that can currently be employed to align sequences and treat indels in subsequent analyses. By covering the main points associated with the generation and analysis of intron data, this review aims to provide a comprehensive introduction to using introns (or any non-coding nuclear data partition) in contemporary phylogenetics.

## Introduction

Non-coding introns are now routinely used in molecular systematics as independent markers (Oakley and Phillips, 1999; van Oppen et al. 2000), or in concert with other gene partitions in an attempt to recover gene trees that are concordant with species trees in plant (Borsch et al. 2003; Guo and Ge, 2004; Oh and Potter, 2005; Shaw et al. 2005; Zhang et al. 2006), fungal (Freeman et al. 2002; Froslev et al. 2005; Cortinas et al. 2006) and animal (Palumbi and Baker, 1994; Prychitko and Moore, 1997; Flynn and Nedbal, 1998; Pitra et al. 2000; Johnson and Clayton, 2000; Rockman et al. 2001; Weibel and Moore, 2002; Rowe and Honeycutt, 2002; Birks and Edwards, 2002; Ericson et al. 2002; Braband et al. 2002; Beltrán et al. 2002; Creer et al. 2003, 2006) molecular phylogenetics. Despite the exponential rise of intron-based molecular genetic studies (Friesen et al. 1997, 1999; Friesen, 2000; Zhang and Hewitt, 2003), a number of genomic, molecular biological, and analytical issues need to be considered during the progression from project conception to data analysis. Factors such as locus selection, paralogy, the occurrence of length variant heterozygotes (LVHs), alignment, insertion/deletion (indel) or gap treatment, and the detection of recombination can all influence how data are generated and analyzed, but such issues are infrequently, or incompletely discussed in empirical studies. Accordingly, this review aims to provide a reference point related to the above issues in order to facilitate an easy introduction to working with introns, or other non-coding data partitions, in molecular phylogenetics.

## What are Introns?

Introns are untranslated gene regions of genomic DNA that are spliced out in the formation of mature RNA molecules and can be conveniently divided into groups, based on their splicing mechanism. Group I and II introns are characterized by different self-splicing mechanisms and are found in some bacterial and organellar genomes (Kelchner, 2000, 2002; Hausner et al. 2006), and group I introns are also found in ribosomal RNAs (rRNAs) of protists and fungal nuclei. Conversely, spliceosomal introns (the most common insertions in eukaryotic nuclear pre-mRNA genes) require a complex of five RNAs and hundreds of proteins, known as the spliceosome, to facilitate intron excision in the formation of mature messenger RNA molecules (Bonen and Vogel, 2001; Roy and Gilbert, 2006). In eukaryotic introns, two types of spliceosome are recognized. The common U2-type splices GT-AG introns, so called because the introns start with 5’ GT and end with 3’ AG dinucleotides, and possess a characteristic pyridimine rich region that precedes the 3’ splice site (Stryer, 1988; Senapathy et al. 1990; Friesen, 2000). The second U12-type, splices the vary rare AT-AC introns, that have a number of dinucleotides at the 3’ end (Belshaw and Bensasson, 2006). Finally, transfer RNA (tRNA) introns are found in eukaryotic nuclei and in Archaea, but are spliced enzymatically using a completely different mechanism to spliceosomal introns (Haugen et al. 2005; Roy and Gilbert, 2006).

Introns have been shown to affect eukaryotic gene expression in a number of ways, including initial transcription, editing, polyadenylation and nuclear export of the pre-mRNA, translation and decay of the mRNA product, in addition to exon shuffling, duplication and alternative splicing of discrete genes (Gasch et al. 1989; Alder et al. 1992; Kirby et al. 1995; Leicht et al. 1995; Prychitko and Moore, 2003; Le Hir et al. 2003). Thus, although introns have clear functional significance, empirical data have shown that they can be considered as neutral markers that possess a number of traits that are desirable for molecular phylogenetics (Friesen, 1997, 2000). Compared to coding regions, the non-coding nature of introns predicts the acquisition of a large number of independent parsimony informative characters from most sites equally, associated with less homoplasy and lower transition: transversion ratios (Slade et al. 1994; Prychitko and Moore, 2000, 2003). Nevertheless, depending on the splicing mechanisms that are involved in the excision process, some classes of introns may possess a mosaic-like structure involving conserved and secondary structure elements, and/or mutational hotspots, that appear to evolve under complex and different evolutionary constraints (e.g. compensating base pair changes) (Borsch et al. 2003; Quandt and Stech, 2005; Quandt et al. 2004). Moreover, it must also be acknowledged that diploid spliceosomal intron alleles have an average effective population size four times that of mtDNA and empirical “ball park” estimations in animals have shown that introns mutate at approximately one quarter the rate of animal mtDNA ([Bibr b93-ebo-03-99][Bibr b94-ebo-03-99]; Creer et al. 2003). Consequently, animal mtDNA haplotypes are expected to coalesce (i.e. become monophyletic) and track recent speciation events more rapidly than intron loci (Moore, 1995; Wiens, 2000).

## Locus Selection and Primer Design

The amplification of introns for interspecific studies is usually facilitated by designing primers that anneal to conserved regions within exons to either side of the target intron (e.g. 50 base pairs (bp) upstream and downstream of the 5’ and 3’ intron splice sites for the forward and reverse primers respectively). This exon-primed, intron-crossing (EPIC) primer design strategy was introduced over ten years ago (Lessa, 1992; Slade et al. 1993), but widely applicable primers such as those that have contributed to the meteoric success of various animal mtDNA genes (Kocher et al. 1989) have yet to be realised (Zhang and Hewitt, 2003; Hughes et al. 2006).

### Empirical testing

Although truly universally applicable primers do not exist, a number of putatively broad, and taxon-specific EPIC primers are now available for potential use in animals (Slade et al. 1993; Palumbi and Baker, 1994; Friesen et al. 1997, 1999; Prychitko and Moore, 1997; Hassan et al. 2002; Jarman et al. 2002; Touriya et al. 2003; Sota and Vogler, 2003; Aitken et al. 2004) and vascular and non-vascular plants, (Shaw et al. 2005; Ishikawa et al. 2002). Thus, one approach that can be utilized to locate a suitable selection of markers, is to assay the performance of numerous primers from existing studies (Creer et al. 2005), or primers that have worked well in related taxa. Using a diverse array of PCR optimization strategies on representatives from a desired phylogenetic range may not result in complete success, but the approach is predicted to identify a subsection of primers that will result in successful amplifications throughout the target genetic group. If amplifications are lacking in a number of taxa for a particular locus, the sequences derived from the successful PCR reactions can, and should where possible, serve as templates for taxon-specific EPIC (Slade et al. 1993) or even intron (I) PIC primer designs (i.e. where the primers are designed within conserved regions of the actual intron).

### Data mining

In August 2005, the amount of sequence data available in the GenBank repository of the National Center for Biotechnology Information website (NCBI – http://www.ncbi.nlm.nih.gov/) exceeded 100 gigabases, i.e. over 100 billion base pairs. A significant proportion of these data will correspond to model organisms and genome sequencing projects, but there is also currently an abundance of annotated whole genomic and mRNA cDNA sequences that can be mined for tailor-made EPIC priming sites in target taxa spanning appropriate phylogenetic ranges. Bioinformatic tools such as Spidey (http://www.ncbi.nlm.nih.gov/Tools/) now make the task easier by aligning one or more mRNA sequences to single genomic sequences. Primers can then be designed using readily available programs such as Primer 3 (Rosen and Skaletsky, 1998) for single sequences, or PriFi (Fredslund et al. 2005) for multiple sequence alignments.

## Dealing with Paralogy

The above approaches primarily rely on the conservation of assumed single copy nuclear exon coding regions for primer design, however, if gene duplication has occurred, the use of degenerate primers can result in the inadvertent amplification of paralogous loci (Tank and Sang, 2001; Archambault and Bruneau, 2004; Pfeil et al. 2004; Meimberg et al. 2006). Obviously, phylogenetic error can arise if paralogs (genes related by duplication) are mistakenly interpreted to be orthologs (genes derived from a single ancestral gene in the last common ancestor of the compared species) when inferring phylogenetic relationships (Sanderson and Shaffer, 2002; Koonin, 2005). There is some hope that paralogous genes can be detected by differences in molecular architecture, e.g. in size, structure, codon usage or base composition (Cotton, 2005), or via the interpretation of tree topologies that are grossly incongruent with widely held perceptions. Alternatively, attempts to overcome paralogy can be achieved via computational algorithms, or by alternative molecular biological strategies. Maddison (1997), Page and Charleston (1997) and Slowinski et al. (1997) described a procedure, termed “gene tree parsimony” (Page, 1998) that employs heuristic searches for species trees that minimizes the weighted sum of gene duplications plus losses (in addition to deep coalescences and lateral transfers) necessary to fit gene family trees to species trees (Slowinski et al. 1997). In addition to visualizing the fit between gene and known species phylogenies, the program GeneTree (Page, 1998) can be used to infer species phylogenies from duplicated genes, whereby the optimal species tree is that in which the gene trees can be embedded with the least cost. Furthermore, maximum likelihood and Bayesian methods that allow probabilistic model incorporation are now being developed for reconciling gene and species trees (Arvestad et al. 2003). Such directions show particular promise in overcoming the conceptual limitations involved in reconciliation approaches reliant solely on the principles of parsimony (Cotton, 2005).

On the other hand, the generation of cDNA libraries and designing one primer in the 3’ untranslated region (3’ UTR) and the other in coding regions has been suggested as a molecular-based method to overcome the amplification of non-orthologous genes. Such an approach aims to exploit the fact that divergence between paralogous loci is likely to occur more rapidly in the 3’UTR compared to adjacent exons. However, the specificity gained by using the exon-3’UTR approach is likely to come at a cost in that priming site substitutions are likely to result in PCR failure as genetic distance increases from the species from which the cDNA was sequenced (Whittall et al. 2006).

It is therefore likely that a combination of experimental, computational and bioinformatic approaches may yield a number of orthologous and potentially phylogenetically useful genes. Once the loci have been chosen, it is important that the introns should then be sequenced across an appropriate broad taxonomic range to ensure that the markers yield sufficient phylogenetic signal (Shaw et al. 2005; Hughes et al. 2006). Testing a large number of primers may seem labor-intensive, but it is certainly a cost-effective strategy. A short investment of time and money at the outset of a project vastly outweighs the disadvantages associated with working with less than optimal markers throughout a genetically disparate range.

### Addressing recombination

A fundamental concept in molecular phylogenetics is that a single phylogeny can be reconstructed from the sequences under study (Posada and Crandall, 2001; Wiuf et al. 2001; Husmeier and McGuire, 2003). Nevertheless, nuclear genes can frequently experience recombination events that can create mosaic genes (Maynard-Smith, 1992) where different regions possess diverse phylogenetic histories (Posada and Crandall, 2001).

One way to avoid the potentially confounding problem of recombination is to use nuclear genes that experience very low rates of recombination, but a more likely solution is to detect recombination events and incorporate the data into models of molecular evolution during analysis, thus facilitating the fuller exploitation of nuclear markers (Zhang and Hewitt, 2003). A number of methods based upon similarity, distance, phylogeny, compatibility/congruence, distribution of substitutions (Posada and Crandall, 2001; Posada, 2002; Posada et al. 2002) and Bayesian approaches (Husmeier and McGuire, 2003) have been developed to detect recombination (a full list of resources for detecting recombination can be found at http://www.umber.embnet.org/~robertson/recombination/index.shtml). Moreover, software such as Recombination Detection Package 2 (RDP2 available at http://darwin.uvigo.es/rdp/rdp.html) combines ten different published methods in an attempt to identify recombinant sequences and recombination breakpoints (Martin et al. 2005). Programs such as RDP2 utilize a range of approaches as empirical tests on simulated data have shown that no single method is likely to be optimal in detecting recombination under all conditions (Posada and Crandall, 2001; Posada, 2002).

Although recombination is an integral part of meiosis, it appears paradoxical that only a limited number of empirical phylogenetic studies have attempted to detect recombination within nuclear gene datasets (Miadlikowska et al. 2003; Printzen et al. 2003; Jarvinen et al. 2004; Devos et al. 2005; Poke et al. 2006). The lack of instances of recombination detection may reflect the comparative infancy of the field of nuclear, as opposed to mtDNA gene-based molecular systematics (but see Piganeau et al. (2004) and Tsaousis et al. (2005) for recent animal mtDNA recombination surveys). Alternatively, relatively derived taxonomic lineages are precluded from recombination detection analyses as most methods have been shown to fail if sequence divergence is less than five percent (Posada and Crandall, 2001; Devos et al. 2005). Still, for datasets reflecting deeper phylogenetic levels, the above recent bioinformatic innovations suggest there are no reasons why recombination detection cannot form an integral part of sequence analyses during phylogeny reconstruction. Depending on the particular scenario, the taxa involved in the recombination event may then either be excluded from the analysis, or included, and the recombination information integrated into phylogeny reconstruction or interpretation.

### Detection, separation and incorporation of LVHs

Diploid non-coding nuclear genes will either be heterozygotic or homozygotic, but it is infrequently reported that heterozygotic introns often differ in length. Heterozygotic introns of the same length are easily recognised in direct sequencing chromatograms as dual peaks of approximately equal intensity occupying the same base position, and can be detected by eye, or using software such as Polyphred (Nickerson et al. 1997). The latter ambiguous sites can be scored as Ns for phylogenetic purposes, but direct sequencing a LVH will result in the apparent corruption of the sequence reaction due to the superimposition of two separate sequence chromatograms occupying the same frame (Mallarino et al. 2005). If a LVH is suspected, many solutions exist to separate the two alleles including using the ‘allele-dropout-effect’, haplotype separation by single strand conformation polymorphism (SSCP) and denaturing gradient gel electrophoresis (DGGE) (Zhang and Hewitt, 2003). For most laboratories dealing with phylogenetic analyses, cloning can provide an easy solution for separating the two alleles, although separating haplotypes with denaturing high performance liquid chromatography (DHPLC) may be the future solution towards resolving LVHs that prove to be difficult to clone, or for high-throughput purposes (Underhill et al. 1996, 1997; Zhang and Hewitt, 2003).

The ocurrence of LVHs was first highlighted by Palumbi and Baker (1994), but a large proportion of intron-based molecular phylogenetic studies do not mention LVHs, and others have attempted but have failed to detect intra-individual length variation (van Oppen et al. 2000; Prychitko and Moore, 2000; Birks and Edwards, 2002). Recently however, studies have detected and incorporated LVHs into phylogenetic frameworks (Beltrán et al. 2002; Sota and Vogler, 2003; Pons et al. 2004; Creer et al. 2006) suggesting that the phenomenon is common within intron loci and should be considered as a matter of course in studies using diploid introns as phylogenetic markers. The analysis of LVHs within a single locus analysis is simple, but multiple-partition total evidence (Kluge, 1989; Nixon and Carpenter, 1996) approaches provide additional challenges. Sota and Vogler, (2003) recently employed an intuitive approach whereby LVHs are simultaneously incorporated as independent terminals in data matrices by duplication of homozygotic loci alongside heterozygotic loci. Therefore, if an individual was homozygotic for locus A and heterozygotic for locus B, the heterozygotic taxon would be represented by two identical allele A sequences (AA) and the two length variant B loci sequences (Bb), and vice versa. If, on the other hand, the individual was heterozygotic for both loci, all four combinations of the LVHs are included in the analyses. This approach may be tractable with a limited number of partitions, but the number of independent terminals (represented by 2*^n^*, where *n* is the number of loci) may prove cumbersome with combinations of multiple heterozygotic loci. An emerging solution to this problem may lie in the Phylogeny of Organisms from Allelic Data (POFAD) algorithm that converts distance matrices of alleles to organismal distance matrices from one or more genes (Joly and Bruneau, 2006), but further independent testing will be needed to confirm or refute its systematic utility.

### Alignment and indel treatment approaches

Non-coding nuclear gene partitions frequently experience diverse indel events that create considerable alignment problems. In order to achieve positional homology, multiple DNA sequences are therefore either aligned “by-eye” or by using a range of algorithms implemented by computer programs such as ClustalX (Thompson et al. 1997), T-Coffee (Notredame et al. 2000), DIALIGN (Morgenstern, 1999), or MUSCLE, that is recommended for large numbers of sequences (Edgar, 2004). Proponents of algorithm-based alignments criticize the subjectivity and lack of repeatability of “by-eye” alignments (Giribet and Wheeler, 1999), although some empirical studies have shown that manual alignments are not significantly worse than computer assisted alignments (Sanchis et al. 2001; Belshaw and Quicke, 2002). On the other hand, the diversity and length of indels experienced in intron partitions frequently cause computer-based alignments to have significant proportions of misaligned taxa or gene regions. The often used and optimal strategy may therefore be to utilize appropriate programs and amend any obviously misaligned regions by hand (Freudenstein and Chase, 2001; Sanchis et al. 2001; Kawakita et al. 2003; Creer et al. 2006). The manual intervention in the latter scenario does introduce non-objectivity, but the complete removal of subjectivity in complex alignments is likely to remain a utopian goal (Lutzoni et al. 2000).

Following alignment, the next step is to decide what to do with the indel data. The classical strategy is to treat alignment gaps as missing data (Kumar et al. 2004; Swofford et al. 1996; Swofford, 1998) and such an approach is attractive if the indel events are minor. Indels however, may represent Hennigian biological events (Archambault and Bruneau, 2004) and often represent a substantial percentage of sequence data in non-coding data partitions. Disregarding gap data may therefore represent the loss of a considerable proportion of phylogenetic signal (Freudenstein and Chase, 2001). In attempt to remedy this situation, a number of approaches have emerged to incorporate gap characters with substitutional data in phylogenetics.

Perhaps the simplest approach is to code gaps as fifth character states (Swofford, 1998). Alternatively, gaps with different start and/or end positions can be replaced (i.e. treat as missing data) with a coded binary matrix (based on presence/absence) that is concatenated and analysed with the normal DNA data. Simmons and Ochoterena, (2000) formalized this approach with the advent of “simple coding” and the software GapCoder (Young and Healy, 2003) facilitates the construction of the binary matrix. A newer approach, called Modified Complex Indel Coding (MCIC) has additionally been developed that aims to maximize the phylogenetic information retained from unambiguously aligned sequences that was previously not utilized by simple coding (Müller, 2006). Modified Complex Indel Coding can be performed using “IndelCoder” within the program SeqState (Müller, 2005).

Introns frequently experience combinations of indels and substitutions that result in areas that cannot be aligned equivocally. Homopolymers, pyridimine rich (both independent, and inclusive of the 3’ splice site (Senapathy et al. 1990)) and A/C rich regions all appear to be commonplace. In order to overcome the homology problems associated with areas of ambiguous alignment, the software INtegrating Ambiguously Aligned SEquences INAASE (Lutzoni et al. 2000) expedites the replacement of these regions with multistate coded characters (step matrices), that are analysed alongside the DNA base characters. Thus, by replacing the area of ambiguous alignment with a step matrix, multistate coding attempts to incorporate unequivocally aligned regions of DNA without violating positional homology (Lutzoni et al. 2000). Nevertheless, Müller (2005) points out that INAASE effectively ignores some characters in delimited multistate regions and does not address the issue of incorporating information from length mutational events in regions for which positional homology has been established. Alternatively, indel coding methods use the information from length mutational events as well as the information from substitutional data in the same region.

Simple coding, MCIC, multistate coding and coding gaps as a fifth character state all rely on posterior coding of indels that are derived from a multiple sequence alignment (Wheeler, 2001). Alternatives lie in fixed-state optimization (Wheeler, 1999) and direct optimization (Wheeler, 1996) that can be executed using the software POY (Wheeler and Gladstein, 2000; for debate, refer to Simmons, 2004 and Kluge and Grant, 2006). Both approaches differ from multiple sequence alignment based methods as the sequence data is not preprocessed, but proceed directly to cladogram optimization (Wheeler, 2001). Fixed-state optimization treats each sequence as a character state, and generates a matrix of transformation costs that relate different states to one another, in a similar fashion to multistate coding (Wheeler, 1999). Alternatively, direct optimization incorporates indel events as additional transformations during the optimization step in tree evaluation instead of trying to reconcile sequence lengths by adding gaps as additional states. Substitutions and indel events are simultaneously minimized and unique alignments are generated for each historical hypothesis (Aagesen, 2005; Hormiga et al. 2003; Wheeler, 2001).

All of the above solutions addressing alignment and gap treatment strategies have been approached using parsimony. Very recently however, a number of methods have emerged that aim to simultaneously infer multiple alignment and construct phylogenetic hypotheses using Bayesian approaches. Lunter et al. (2005) and Fleissner et al. (2005) have used the TKF1 (Thorne et al. 1991) and the TKF2 (Thorne et al. 1992) models respectively. The TKF1 model treats indels as independent single base pair events, whereas the TKF2 model permits non-nested and non-overlapping indels of several base pairs in length. Alternatively, Redelings and Suchard, (2005) have adopted a novel model and algorithm that allows multiple base pair, overlapping and nested indels, accommodating all homology structures.

Thus, indel treatment strategies can be conveniently split into static vs. dynamic and parsimony vs. model-based approaches, but it is also pertinent to acknowledge that any method that that treats indel characters as independent data points (e.g. fifth state, POY and Lunter et al.’s (2005) Bayesian approach) disregards any knowledge concerning the biological mechanisms underlying indel evolution. Indel mutation processes are unlikely to arise from the same mutational mechanisms as substitutional data (Pons and Vogler, 2006). Smaller gaps (1–30 b.p.) are hypothesized to result from slipped-strand mispairing while it is thought that larger gaps (>30 b.p.) are caused by unequal crossing over or due to transposition (Giribet and Wheeler, 1999; Freudenstein and Chase, 2001; Li, 1997). Therefore, 1—*n* b.p. gaps are often unlikely to represent 1—*n* independent mutation events and methods that treat gaps as independent characters (ie base pair by base pair) can significantly overweight larger indels, compared to smaller, equivalent indel events and can therefore generate innaccurate trees (Lutzoni et al. 2000; Freudenstein and Chase, 2001; Creer et al. 2006). According to this logic, simple coding, MCIC and the Bayesian approaches of Fleissner et al. (2005) and Redelings and Suchard (2005), differ from all the other approaches by treating indels, regardless of length, as independent events.

Finally, following a diverse combination of alignment and indel treatment approaches, a decision must be made regarding which phylogenetic hypothesis most accurately represents evolutionary history (Giribet and Wheeler, 1999; Sanchis et al. 2001). It is widely acknowledged that congruence among datasets provides an accurate estimate of phylogeny (Miyamoto and Fitch, 1995; Giribet and Wheeler, 1999; Wheeler, 2001; Sanchis et al. 2001). Thus, if no independent congruence measures are available, all hypotheses can be presented and shared topologies discussed regarding data-dependent consensus (Arnedo et al. 2004). If however, independent but incompatible datasets are available (e.g. morphology, other genes omitting significant indels, or extremely large datasets) taxonomic congruence can be used as a measure favoring treatments that maximize phylogenetic consensus (Giribet and Wheeler, 1999; Cognato and Vogler, 2001; Giribet, 2001; Belshaw and Quicke, 2002). Alternatively, if multiple data partitions are compatible, character congruence, often measured by incongruence length difference (ILD, Mickevich and Farris (1981)), can be used to objectively assess treatment associated homoplasy through simultaneous analyses (Giribet and Wheeler, 1999; Wheeler, 2001).

In summary, working with introns, or other non-coding nuclear partitions is not as straightforward as working with organellar data (Sang, 2002). Bioinformatic approaches, or assaying a large number of EPIC primers may be required to locate the most appropriate markers for non-model organisms. PCRs may need more optimization due to the degeneracy of the primers involved, and/or the single copy nature of nuclear targets, and direct sequencing may not be possible if LVHs are discovered. Once the data has been generated from orthologous loci, recombination checks can now be routinely performed and a range of parsimony and model-based analytical innovations are also available regarding alignment and the treatment of indel data. Given the growing reliance of the molecular systematic community on non-coding DNA, a key goal that remains is to identify which analytical methods most accurately recover phylogenetic history (Wheeler, 1996; Giribet and Wheeler, 1999; Simmons and Ochoterena, 2000; Lutzoni et al. 2000; Fleissner et al. 2005; Creer et al. 2006). It is therefore important that further testing of all the methods is performed on simulated and empirical datasets to establish which strategies are optimal when using introns or non-coding nuclear partitions as phylogenetic markers.
